# RELATIONSHIP BETWEEN CARE HOME STAFFING AND QUALITY OF CARE: A MIXED-METHODS APPROACH

**DOI:** 10.1093/geroni/igz038.908

**Published:** 2019-11-08

**Authors:** Karen Spilsbury, Andy Charlwood, Danat Valizade, Kirsty Haunch

**Affiliations:** 1 University of Leeds, Leeds, United Kingdom; 2 University of Leeds, Leeds, England, United Kingdom

## Abstract

Beyond broad recognition that ‘staff influence quality’, little is known about the care home workforce and its relationship to quality. Our study examines this relationship for the first time in the UK. Quality is a complex, contested and dynamic concept: we have operationalised this concept using data collected at national and organisational levels to measure quality, as well as considering the views of quality of different stakeholders. We will present interim findings from this study’s work packages (due to complete in July 2020), including: (1) our review which has developed theoretical explanations of how, why and in what circumstances staffing promotes quality for residents and relatives; and (2) our observational study using routine administrative data sets at national and provider level. Key findings include the importance of experience and stability of care hoe staff, as well as how care is delivered (individual commitment, team reciprocity and organisational mandate).

